# Assessing Nonalcoholic Fatty Liver Disease in Type 2 Diabetes Patients at a Tertiary Hospital, Addis Ababa, Ethiopia

**DOI:** 10.1155/jdr/5555842

**Published:** 2025-08-23

**Authors:** Edomias Adyamseged Berhe, Rediet Ambachew Sema, Yididya Mehari Tesfaye, Abel Andargie Berhane, Mikale Dawit, Ephrem Mamo Gebrehiwot, Subah Abderehim Yesuf

**Affiliations:** ^1^Department of Internal Medicine, Yekatit 12 Hospital Medical College, Addis Ababa, Ethiopia; ^2^Department of Epidemiology, School of Public Health, Yekatit 12 Hospital Medical College, Addis Ababa, Ethiopia; ^3^Family Medicine Unit, St. Paul's Hospital Millennium Medical College, Addis Ababa, Ethiopia

**Keywords:** Ethiopia, magnitude, nonalcoholic fatty liver disease, Type 2 diabetes mellitus

## Abstract

**Background:** Nonalcoholic fatty liver disease (NAFLD) is an emerging global health concern that commonly occurs in patients with Type 2 diabetes mellitus (T2DM). However, there is limited literature on the epidemiology of NAFLD among adults with T2DM in Ethiopia. Therefore, this study is aimed at assessing the prevalence of NAFLD and its associated factors in patients with T2DM attending Yekatit 12 Hospital Medical College in Addis Ababa, Ethiopia.

**Methods:** An institution-based, cross-sectional study was conducted. Data were collected using a pretested, structured data collection tool. All eligible consecutive patients diagnosed with T2DM were enrolled in this study. Data were entered into Microsoft Excel 2016 and analyzed using the Statistical Package for the Social Sciences (SPSS) Version 26. Descriptive statistics were used to summarize the data. Multivariable logistic regression was performed to identify associations between dependent and independent variables by calculating odds ratios with corresponding 95% confidence intervals. A *p* value of < 0.05 was considered statistically significant.

**Results:** A total of 211 patients were enrolled in the study. Females (108; 51.2%) slightly predominated, and the mean (standard deviation) age of patients was 56.2 (11.0) years. Fatty liver was detected in 102 patients, representing a prevalence of 48.3% (95% CI: 42%–55%). Mild, moderate, and severe NAFLD accounted for 19.0%, 24.6%, and 4.7%, respectively. Female sex (AOR = 2.27 [95% CI: 1.17, 4.41]), obesity (AOR = 6.13 [95% CI: 2.15, 17.46]), borderline serum triglyceride levels (AOR = 3.22 [95% CI: 1.36, 7.58]), and high serum triglyceride levels (AOR = 2.29 [95% CI: 1.03, 5.10]) were significantly associated with the presence of NAFLD.

**Conclusions:** NAFLD is highly prevalent among patients with T2DM in this Ethiopian cohort. Female sex, obesity, and elevated serum triglyceride levels are significant risk factors. These findings highlight the urgent need to address the silent epidemic of NAFLD among adults with T2DM in Ethiopia and emphasize the importance of educating patients on adopting healthy lifestyles to reduce the incidence of this condition.

## 1. Introduction

Nonalcoholic fatty liver disease (NAFLD) is characterized by the presence of excessive fat accumulation in the liver parenchyma, identified either through imaging or histological examination, in the absence of alternative causes such as significant alcohol consumption, steatogenic medications, or other medical conditions [1–3]. Histologically, NAFLD represents a spectrum of disease ranging from simple steatosis to more advanced forms involving hepatic complications such as hepatitis, fibrosis, cirrhosis, and, in some cases, hepatocellular carcinoma [4, 5]. In the diabetic population, NAFLD is alternatively termed metabolic dysfunction–associated fatty liver disease (MAFLD), defined by evidence of hepatic steatosis along with one of the following: overweight/obesity, Type 2 diabetes mellitus (T2DM), or metabolic dysregulation [6].

NAFLD is further classified into nonalcoholic fatty liver (NAFL) and nonalcoholic steatohepatitis (NASH). NAFL is characterized by ≥ 5% hepatic steatosis without evidence of hepatocellular injury, such as hepatocyte ballooning. In contrast, NASH is a distinct subtype marked by ≥ 5% hepatic steatosis accompanied by inflammation and hepatocyte injury, including ballooning, with or without fibrosis [7].

As a standalone clinicopathological entity, NAFLD has emerged as a significant public health concern [8, 9], affecting approximately one-quarter of the global population [10, 11]. Its prevalence varies geographically, with the highest rates observed in the Middle East (32%), followed by South America (30%), Asia (27%), North America (24%), Europe (24%), and Africa (13%) [12, 13]. Moreover, its prevalence has risen dramatically over recent years, making it the most common cause of chronic liver disease worldwide [4, 8, 14].

The global prevalence of diabetes mellitus (DM) has reached pandemic levels, affecting 10.5% (536.6 million) of the world's population in 2021, with projections estimating an increase to 12.2% (783.2 million) by 2045 [15]. T2DM accounts for over 90% of all DM cases [16]. Reflecting this global trend, the prevalence of T2DM in Ethiopia is also notably high, affecting 6.5% of the population [17]. NAFLD is strongly associated with T2DM [18, 19], resulting in a substantially higher prevalence of NAFLD among diabetic patients compared to the general population [20]. The coexistence of NAFLD and T2DM often leads to synergistic effects that complicate clinical management and worsen outcomes [21].

Various factors have contributed to the dramatic global rise in T2DM incidence and prevalence [15, 22]. This escalating health crisis significantly impacts both lifespan and quality of life [23]. Particularly concerning is the burden of T2DM in low- and middle-income countries, where suboptimal glycemic control remains widespread [22, 24].

The growing impact of T2DM is especially pronounced in sub-Saharan Africa, including Ethiopia [25], where healthcare systems face challenges such as limited availability and affordability of medications, shortages of trained healthcare professionals, and overall inadequate infrastructure [26].

With the increasing prevalence of diabetes and obesity, the incidence of NAFLD and its complications is expected to rise correspondingly [10, 11, 27, 28]. NAFLD has become a leading cause of morbidity and mortality among patients with T2DM due to hepatic complications like cirrhosis and hepatocellular carcinoma, as well as extrahepatic conditions including cardiovascular disease, chronic kidney disease, colorectal cancer, endocrinopathies, and osteoporosis [5, 29]. Additionally, NAFLD imposes a substantial and growing economic burden, with estimated annual costs reaching $103 billion in the United States alone [30].

The prevalence of NAFLD is often underestimated because it is typically asymptomatic and frequently overlooked in clinical practice [31, 32]. In African populations, data on the burden and spectrum of NAFLD among high-risk groups remain scarce [33]. This gap is evident in Ethiopia, where no specific NAFLD-related guidelines exist, and over half (55.3%) of chronic liver diseases are of cryptogenic origin [34]. NAFLD is also a primary cause of unexplained elevated liver enzymes in this setting [35]. Given this context, the present study was conducted to assess the prevalence of NAFLD and its associated factors in patients with T2DM at the Yekatit 12 Hospital Medical College (Y12HMC), Addis Ababa, Ethiopia.

## 2. Methods

### 2.1. Study Setting, Design, and Period

This institution-based, cross-sectional study was conducted at the medical referral and outpatient unit of Y12HMC in Addis Ababa, Ethiopia. Y12HMC is a tertiary public hospital that provides a wide range of clinical services. The medical referral and outpatient unit offers specialized care to more than 2000 patients diagnosed with T2DM. This unit delivers comprehensive diabetic care and is staffed by subspecialists, resident physicians, general practitioners, and trained nurses. Data collection for the study took place from November 1, 2023, to February 29, 2024.

### 2.2. Population

The source population for this study included all patients with T2DM who had regular follow-up at Y12HMC in Addis Ababa, Ethiopia. The study population consisted of consecutively selected T2DM patients attending regular follow-up at the medical referral and outpatient units of the hospital who met the eligibility criteria. Inclusion criteria were adults aged 18 years or older, diagnosed with T2DM by their treating physician, and under regular follow-up for at least 2 months at the time of data collection. Exclusion criteria included patients with a self-reported history of excessive alcohol consumption; documented viral, alcoholic, drug-induced, or autoimmune hepatitis; chronic liver disease; use of steatogenic drugs (such as tamoxifen, amiodarone, or methotrexate) within the past 3 months; thyroid dysfunction; hyperuricemia or gout; gestational diabetes; chronic kidney disease; and pregnancy.

### 2.3. Sample Size Determination and Sampling Technique

The sample size was determined a priori using Tabachnick and Fidell's formula, which recommends a minimum of 50 plus eight times the number of independent variables (i.e., participants > 50 + 8 × independent variables) [36]. Based on 19 variables previously associated with NAFLD in patients with T2DM, this calculation indicated a minimum sample size of 202 participants.

Although 2000 adult T2DM patients were being followed at referral clinics, only about 300 were expected to attend during the study period. Given the likelihood of substantial exclusions due to strict eligibility criteria, the researchers adopted a consecutive enrollment strategy to maximize statistical power by including all eligible participants.

### 2.4. Data Collection and Variables

In this study, the presence of NAFLD was designated as the dependent variable. The independent variables included a range of clinicodemographic factors: age, sex, place of residence, body mass index (BMI), smoking status, duration of diabetes, and hypertension status. Additional independent variables encompassed biochemical and pharmacological characteristics, such as glycated hemoglobin (HbA1c), fasting blood glucose levels, liver and lipid biochemistry, and the use of lipid-lowering agents, antihypertensive medications, and antidiabetic treatments.

Data were extracted from electronic medical records (EMRs) using a structured tool adapted from prior studies [37–39]. Trained nurses collected anthropometric measurements, including BMI (calculated as weight in kilograms divided by height in meters squared), during clinic visits. For cases with incomplete records, patients were contacted virtually and instructed to fast prior to their scheduled laboratory or imaging appointments. Two healthcare professionals underwent standardized training to ensure strict adherence to the study protocol.

### 2.5. Data Quality Management

To ensure high data quality, the data collection tool was developed through an iterative refinement process. Its design was informed by a comprehensive review of relevant literature and related studies [32, 37–39]. A pretest involving 32 patients was conducted, leading to necessary adjustments to the tool. Prior to data collection, two data collectors received targeted training on the tool's fundamentals and ethical considerations. Throughout the data collection phase, the principal investigator provided continuous supervision to ensure completeness, clarity, and accuracy of the data. Completed checklists were audited daily for consistency and completeness, with any ambiguous or incomplete entries excluded from the final analysis.

### 2.6. Statistical Analysis

Data entry, coding, and cleaning were conducted using Microsoft Excel 2016. The cleaned dataset was subsequently exported to the Statistical Package for the Social Sciences (SPSS) Version 26 (IBM Corp., Armonk, NY, United States) for statistical analysis. Descriptive statistics were used to summarize basic patient characteristics. Binary logistic regression was performed to examine associations between variables. Variables with a *p* value ≤ 0.25 in the bivariable logistic regression were included in the multivariable logistic regression model to calculate adjusted odds ratios with 95% confidence intervals. Model fit was assessed using the Hosmer–Lemeshow goodness-of-fit test. Variables with *p* values ≤ 0.05 were considered statistically significant.

### 2.7. Operational Definitions

NAFLD was defined based on the degree of liver stiffness or steatosis as measured by ultrasonography or previously documented abdominal computed tomography (CT) scans or FibroScan [40]. Grade 1 (mild) represented a light increase in liver echogenicity with normal visualization of intrahepatic vessels and no posterior acoustic attenuation, involving 5%–33% of hepatocytes. Grade 2 (moderate) was defined by a moderate increase in liver echogenicity, partial obscuration of vessels, and early posterior acoustic attenuation, affecting 34%–66% of hepatocytes. Grade 3 (severe) denoted a diffuse increase in echogenicity, absence of visible vessels, and marked posterior acoustic attenuation, with more than 66% of hepatocytes involved [40, 41].

## 3. Results

### 3.1. Background Characteristics of the Study Patients

A total of 211 patients with T2DM who regularly attended the medical outpatient clinic at Y12HMC in Addis Ababa, Ethiopia, were included in this study. Females slightly outnumbered males, comprising 108 participants (51.2%). The patients' ages ranged from 30 to 89 years, with a mean (± standard deviation) of 56.2 ± 11.0 years. More than two-thirds of the participants (142; 67.3%) were over 50 years old at the time of the study. Nearly all patients (210; 99.5%) were residents of Addis Ababa. Regarding BMI, 91 patients (48.8%) had a normal BMI, while 85 (40.8%) were classified as overweight. Only three patients (1.4%) reported being smokers (Table 1).

### 3.2. Clinical and Pharmacological Details of Study Patients

With respect to the clinical profile of the studied patients, 91 (43.1%) had a disease duration ranging from 5 to 10 years, while 82 (38.9%) had a disease duration of less than 5 years. More than two-thirds (144, 68.2%) were taking oral hypoglycemic agents, whereas 41 (19.4%) were on both injectable insulin and oral hypoglycemic agents. Metformin monotherapy was prescribed to 75 (35.5%) patients, while 64 (30.3%) were receiving a combination of metformin and sulfonylurea. Additionally, 117 (55.5%) of the patients were hypertensive, with 115 (54.5%) receiving antihypertensive medications. The majority (117, 55.5%) were also taking lipid-lowering agents (Table 2).

### 3.3. Biochemical Details of Study Patients

Then, 72 patients (34.1%) achieved optimal glycemic control, with preprandial blood glucose levels within the target range of 80–130 mg/dL. Among the 75 patients with recorded HbA1c levels from the preceding 3 months, 61 (81.3%) exhibited elevated values (Table 3).

Elevated alanine transaminase (ALT) and aspartate aminotransferase (AST) levels were observed in 25 patients each (11.8% for both), with an AST-to-ALT ratio > 1 in 93 patients (44.1%). While total bilirubin was elevated in seven (3.3%) of the patients, about one-fourth (*n* = 51, 24.2%) of the patients had elevated direct bilirubin levels. The serum levels of total cholesterol and triglycerides were highly elevated in 16 (7.6%) and 63 (29.9%) of the patients, respectively. Besides, high-density lipoprotein was low in 155 (73.5%), and low-density lipoprotein was elevated in 132 (62.6%) of the patients (Table 3).

### 3.4. Magnitude of NAFLD Among the Study Patients

**Figure 1:**
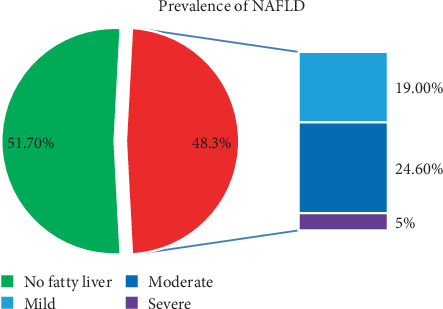
Magnitude of NAFLD among patients with Type 2 diabetes mellitus having regular follow-up at Yekatit 12 Hospital Medical College, Addis Ababa, Ethiopia (*n* = 211).

In this study, the magnitude of NAFLD among patients with T2DM was 48.3% (95% CI: 42%–55%). Specifically, 52 patients (24.6%) exhibited moderate NAFLD, while 40 patients (19.0%) had mild NAFLD. In contrast, only 10 patients (4.7%) were found to have severe NAFLD based on ultrasonographic findings (Figure 1).

### 3.5. Factors Associated With NAFLD

After controlling for variables with *p* < 0.25 in bivariable regression, this study found that women had higher odds of developing NAFLD compared to men (AOR = 2.27 [95% CI: 1.17, 4.41]). Similarly, obese patients (BMI ≥ 30) were significantly more likely to have NAFLD than those with a normal BMI (AOR = 6.13 [95% CI: 2.15, 17.46]). Additionally, patients with borderline or high serum triglyceride levels were more likely to have NAFLD than those with normal triglyceride levels, with adjusted odds ratios of 3.22 (95% CI: 1.36, 7.58) and 2.29 (95% CI: 1.03, 5.10), respectively. The Hosmer–Lemeshow goodness-of-fit test demonstrated adequate model fit (*p* = 0.300), indicating no significant deviation between observed and predicted outcomes (Table 4).

## 4. Discussion

This study evaluated the prevalence of NAFLD among patients with T2DM attending clinics in Addis Ababa, Ethiopia. NAFLD was diagnosed using abdominal ultrasound imaging, revealing a prevalence of 48.3% among participants. This rate is consistent with findings from India (51.3%) and Iran (55.8%) [31, 42] but is somewhat lower than reported prevalences in Northeast Ethiopia (58.4%) and China (58.67%) [32, 43].

In contrast, the prevalence observed in this study is substantially lower than the 80.4% reported in Jordan and the 68.1%–73% range documented in Southwest Ethiopia, Bahrain, and Southwestern Saudi Arabia, where abdominal ultrasound was also employed for diagnosis [38, 39, 44, 45]. Conversely, it exceeds the 42% prevalence reported in a Brazilian population assessed by ultrasound [46].

Differences in prevalence rates may be attributed to variations in diagnostic methods, technician expertise, clinical characteristics of the diabetic populations, and medication use. For instance, the Saudi Arabian study reported a predominance of males (66.1%), whereas this study included less than half males (47.3%) [45]. Additionally, obesity prevalence differed markedly, with only 17.3% of participants classified as obese in this study compared to 65.9% in the Jordanian study. The use of lipid-lowering agents was also higher in the Bahraini study, where 77.2% of participants were on statins, compared to 63.3% in this cohort [38, 44].

This study found that women were more likely to have NAFLD than men, consistent with findings from Northeast Thailand, where postmenopausal women aged 56–60 exhibited higher NAFLD prevalence [47]. Although this observation contrasts with some previous reports [31, 38], it aligns with emerging evidence suggesting that estrogen deficiency, relative androgen excess, and decreased sex hormone–binding globulin in postmenopausal women increase NAFLD risk. Also, age-related changes in adipose tissue distribution, with increased visceral fat, further elevate NAFLD risk in this group [47, 48].

Obesity was significantly associated with NAFLD in this cohort, corroborating studies from Brazil and Saudi Arabia [45, 46]. Obesity promotes ectopic fat accumulation, adipose tissue dysfunction, and elevated proinflammatory cytokines, all of which contribute to NAFLD pathogenesis. Furthermore, obesity disrupts the balance of hepatic fatty acid uptake and synthesis, leading to excessive intrahepatic triglyceride accumulation characteristic of NAFLD [49]. Notably, T2DM itself is an independent and synergistic risk factor for NAFLD, mediated through mechanisms such as insulin resistance and metabolic dysfunction [50, 51].

Hypertriglyceridemia was independently associated with NAFLD, consistent with previous findings from Ethiopia [39] and Jordan [44]. It contributes to NAFLD pathogenesis by promoting hepatic fat accumulation amid altered mitochondrial function and complex pathophysiological mechanisms [52].

In general, in line with previous reports, the results of this study underscore that lifestyle modifications—particularly weight loss through diet and exercise—remain the cornerstone of NAFLD management, with pharmacologic treatments targeting lipid abnormalities and glycemic control serving as adjunct therapies [53–55].

Structured interventions combining hypocaloric diets with both aerobic and resistance exercise effectively reduce intrahepatic fat, improve insulin sensitivity, and decrease cardiovascular and metabolic risks in this vulnerable population [54–56]. Adoption of a Mediterranean-style diet—characterized by low intake of refined carbohydrates and sugars and high in monounsaturated and Omega-3 fatty acids—has demonstrated benefits in reducing liver fat independently of weight loss, as well as improving metabolic parameters relevant to NAFLD and T2DM [57, 58].

Both aerobic and resistance exercises decrease intrahepatic fat and improve glycemic control by enhancing skeletal muscle metabolism, which is particularly advantageous for patients resistant to dietary interventions alone [54, 59]. Achieving a weight loss of at least 7%–10% improves hepatic inflammation and fibrosis, while weight loss exceeding 10% is associated with fibrosis regression and potential modification of diabetes progression or remission [54, 58]. Finally, statins and other lipid-lowering agents play a critical role in managing hypertriglyceridemia, thereby reducing cardiovascular risk and potentially improving liver outcomes in this population [60].

The study focused on patients with T2DM attending clinics in Ethiopia, providing valuable insights into the prevalence and associated factors of NAFLD within this specific population. This targeted approach enhances our understanding of the relationship between NAFLD and T2DM, which can inform the development of tailored interventions and clinical management strategies. By addressing a significant research gap, the study contributes to the limited data available on NAFLD in Ethiopia and its association with T2DM, thereby establishing a foundation for future research and intervention efforts in similar contexts.

However, several limitations should be acknowledged. First, ultrasound, while noninvasive and accessible, has limited sensitivity for detecting mild steatosis and cannot accurately quantify hepatic fat or assess fibrosis. This may have led to an underestimation of NAFLD prevalence in our cohort. Again, the reliance on ultrasound could result in misclassification, particularly in patients with early or mild disease, and may affect the reported prevalence and severity distribution. Second, more advanced diagnostic methods such as magnetic resonance imaging (MRI), transient elastography (FibroScan), or liver biopsy were not utilized to confirm NAFLD diagnosis or assess disease severity. Third, the study was conducted at a single-center diabetic clinic, which may introduce selection bias. As a result, while our hospital is a major tertiary referral center in Addis Ababa, the design may limit generalizability to all Ethiopian patients with T2DM, especially those in rural or primary care settings. Finally, the cross-sectional design of the study limits the ability to infer causal relationships between NAFLD and its associated factors.

## 5. Conclusions

The study found a high prevalence of NAFLD among diabetic patients, mostly mild to moderate cases. Key factors linked to NAFLD included female sex, obesity, and hypertriglyceridemia. These results underscore the importance of routine NAFLD screening in patients with T2DM, especially women and those with obesity or elevated triglycerides. Early detection and treatment of modifiable risks can significantly lower NAFLD complications in this high-risk group. Healthcare providers should emphasize lifestyle changes focused on weight loss and managing lipid levels to prevent disease progression. Local health policies should support targeted screening using tailored algorithms for diabetic populations. Future research must involve multicenter studies to confirm these findings. Additionally, future studies in Ethiopia should incorporate advanced modalities such as FibroScan, magnetic resonance imaging–proton density fat fraction (MRI-PDFF), or liver biopsy when feasible to improve diagnostic accuracy and staging.

## Figures and Tables

**Table 1:** Background characteristics of patients with Type 2 diabetes mellitus having regular follow-up at Yekatit 12 Hospital Medical College, Addis Ababa, Ethiopia (*n* = 211).

**Variable**	**Frequency**	**Percent (%)**
Age category		
≤ 40 years	20	9.5
41–50 years	49	23.2
51–60 years	72	34.1
> 60 years	70	33.2
Mean (standard deviation)	56.2 (11.0)	
Sex		
Male	103	48.8
Female	108	51.2
Body mass index		
Normal (18.5–24.9)	91	43.1
Overweight (25–29.9)	85	40.8
Obese (≥ 30)	34	16.1
Cigarette smoking		
Yes	3	1.4
No	208	98.6

**Table 2:** Clinical and pharmacological characteristics of patients with Type 2 diabetes mellitus having regular follow-up at Yekatit 12 Hospital Medical College, Addis Ababa, Ethiopia (*n* = 211).

**Variable**	**Frequency**	**Percent (%)**
Disease duration		
< 5 years	82	38.9
5–10 years	91	43.1
> 10 years	38	18.0
Antidiabetic agent route		
Orals	144	68.2
Injectable	26	12.3
Both	41	19.4
Antidiabetic agent		
Metformin	75	35.5
Metformin + sulfonylureas	64	30.3
Metformin + insulin	26	12.3
Insulin	38	18.0
Metformin + sulfonylureas + SGLT2i	4	1.9
Metformin + SGLT2i	4	1.9
History of hypertension		
Yes	117	55.5
No	94	44.5
Antihypertensive medication		
Yes	115	54.5
No	96	45.5
Lipid-lowering agent		
Yes	117	55.5
No	94	44.5

Abbreviation: SGLT2i, sodium–glucose cotransporter 2 inhibitor.

**Table 3:** Biochemical details of patients with Type 2 diabetes mellitus having regular follow-up at Yekatit 12 Hospital Medical College, Addis Ababa, Ethiopia (*n* = 211).

**Variable**	**Frequency**	**Percent (%)**
Preprandial blood glucose		
80–130 mg/dL	72	34.1
> 130 mg/dL	139	65.9
Glycated hemoglobin (*n* = 75)		
< 7%	14	18.7
≥ 7%	61	81.3
Alanine transaminase (ALT)		
Normal	170	80.6
Elevated	41	19.4
Aspartate aminotransferase (AST)		
Normal	186	88.2
Elevated	25	11.8
AST-to-ALT ratio		
≤ 1	118	55.9
> 1	93	44.1
Total bilirubin		
Normal	204	96.7
Elevated	7	3.3
Direct bilirubin		
Normal	160	75.8
Elevated	51	24.2
Total cholesterol		
Normal	175	82.9
Borderline	20	9.5
High	16	7.6
HDL cholesterol		
Normal	56	26.5
Abnormal	155	73.5
LDL cholesterol		
Normal	79	37.4
Elevated	132	62.6
Triglyceride cholesterol		
Normal	105	49.8
Borderline	43	20.4
High	63	29.9

Abbreviations: HDL, high-density lipoprotein; LDL, low-density lipoprotein.

**Table 4:** Factors associated with NAFLD among patients with Type 2 diabetes mellitus having regular follow-up at Yekatit 12 Hospital Medical College, Addis Ababa, Ethiopia.

**Variable**	**NAFLD**		**COR (95% CI)**	**AOR (95% CI)**	**p** ** value**
	**Present**	**Absent**			
Sex					
Male	39 (37.9)	64 (62.1)	1	1	—
Female	63 (58.3)	45 (41.7)	2.30 (1.32, 3.99)	2.27 (1.17, 4.41)⁣^∗^	**0.016**
Age category					
≤ 40 years	12 (60.0)	8 (40.0)	2.39 (0.86, 6.60)	1.95 (0.56, 6.77)	0.291
41–50 years	27 (55.1)	22 (44.9)	1.96 (0.93, 4.10)	1.34 (0.54, 3.37)	0.527
51–60 years	36 (50.0)	36 (50.0)	1.59 (0.82, 3.10)	1.61 (0.74, 3.51)	0.227
> 60 years	27 (38.6)	43 (61.4)	1	1	—
Body mass index					
18.5–24.9	35 (38.5)	56 (61.5)	1	1	**—**
25–29.9	43 (50.0)	43 (50.0)	1.60 (0.88, 2.91)	1.36 (0.69, 2.69)	0.369
≥ 30	24 (70.6)	10 (29.4)	3.84 (1.64, 8.98)	6.13 (2.15, 17.46)⁣^∗∗^	**0.001**
Antihypertensive medication					
Yes	50 (43.5)	65 (56.5)	1	1	—
No	52 (54.2)	44 (45.8)	1.54 (0.89, 2.65)	1.47 (0.76, 2.86)	0.256
Antidiabetic agent route					
Orals	75 (52.1)	69 (47.9)	1.26 (0.63, 2.52)	1.77 (0.77, 4.04)	0.178
Injectable	8 (30.8)	18 (69.2)	0.52 (0.18, 1.45)	0.44 (1.12, 5.21)	0.185
Both	19 (46.3)	22 (53.7)	1	1	—
Preprandial blood glucose					
≤ 130 mg/dL	29 (40.8)	43 (59.2)	1	1	—
> 130 mg/dL	73 (52.5)	66 (47.5)	1.64 (0.92, 2.92)	1.68 (0.84, 3.36)	0.139
Alanine transaminase					
Normal	76 (44.7)	94 (55.3)	1	1	—
Elevated	26 (63.4)	15 (36.6)	2.14 (1.06, 4.33)	2.14 (0.89, 5.17)	0.091
AST-to-ALT ratio					
≤ 1	62 (52.5)	56 (47.5)	1.47 (0.85, 2.54)	1.50 (0.78, 2.90)	
> 1	40 (43.0)	53 (57.0)	1	1	0.225
Total cholesterol					—
Normal	80 (45.7)	95 (54.3)	1	1	
Borderline	12 (66.7)	8 (33.3)	1.78 (0.69, 4.57)	0.87 (0.27, 2.76)	0.810
High	10 (62.5)	6 (37.5)	1.98 (0.69, 5.68)	1.06 (0.32, 3.44)	0.928
Triglyceride cholesterol					
Normal	37 (35.2)	68 (64.8)	1	1	—
Borderline	29 (67.4)	14 (32.6)	3.81 (1.79, 8.08)	3.22 (1.36, 7.58)⁣^∗∗^	**0.008**
High	36 (57.1)	27 (42.9)	2.45 (1.29, 4.65)	2.29 (1.03, 5.10)⁣^∗^	**0.042**

*Note:* Only variables with *p* value < 0.25 in the bivariable regression are depicted here. 1: reference category. The bold entries represent the exact significance values (*p* values) themselves.

⁣^∗^* p* <0.05 and ⁣^∗∗^*p* <0.01.

## Data Availability

The data that support the findings of this study are available on request from the corresponding author. The data are not publicly available due to privacy or ethical restrictions.

## Nomenclature

ALT: alanine aminotransferase
AOR: adjusted odds ratio
AST: aspartate aminotransferase
BMI: body mass index
COR: crude odds ratio
HS: hepatic steatosis
LDL: low-density lipoprotein
HDL: high-density lipoprotein
NAFL: nonalcoholic fatty liver
NAFLD: nonalcoholic fatty liver disease
NASH: nonalcoholic steatohepatitis
SPSS: Statistical Package for Social Sciences
T2DM: Type 2 diabetes mellitus
Y12HMC: Yekatit 12 Hospital Medical College
